# 3,6-Dibromo-9-(4-chloro­benz­yl)-9*H*-carbazole

**DOI:** 10.1107/S1600536808042815

**Published:** 2009-01-08

**Authors:** Jian-lan Cui, Mei Duan, Liu-qing Cai

**Affiliations:** a School of Chemical Engineering and Environment, North University of China, Taiyuan 030051, People’s Republic of China; b Shanxi Provincial People’s Hospital, Taiyuan 030012, People’s Republic of China

## Abstract

The title compound, C_19_H_12_Br_2_ClN, was synthesized by *N*-alkyl­ation of 1-chloro-4-(chloro­meth­yl)benzene with 3,6-dibromo-9*H*-carbazole. The carbazole ring system is essentially planar (mean deviation of 0.028 Å) and makes a dihedral angle of 74.6 (3)° with the plane of the benzene ring.

## Related literature

For the pharmaceutical properties of carbazoles, see: Buu-Hoï & Royer (1950 ▶); Caulfield *et al.* (2002 ▶); Harfenist & Joyner (1983 ▶); Harper *et al.* (2002 ▶). For bond length data, see: Allen *et al.* (1987 ▶). For the synthesis of the title compound, see: Duan *et al.* (2005*a* ▶,*b* ▶); Smith *et al.* (1992 ▶). For related literature, see: Borzatta & Carrozza (1991 ▶). For a related compound, see: Cui *et al.* (2009 ▶).
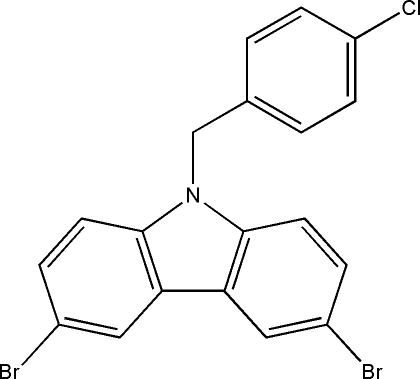


## Experimental

### 

#### Crystal data

C_19_H_12_Br_2_ClN
*M* *_r_* = 449.57
Orthorhombic, 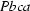

*a* = 8.7673 (18) Å
*b* = 16.732 (3) Å
*c* = 21.574 (4) Å
*V* = 3164.8 (10) Å^3^
*Z* = 8
Mo *K*α radiation
μ = 5.29 mm^−1^
*T* = 113 (2) K
0.06 × 0.02 × 0.02 mm

#### Data collection

Rigaku Saturn diffractometer
Absorption correction: multi-scan (*CrystalClear*; Rigaku/MSC, 2005 ▶) *T*_min_ = 0.742, *T*_max_ = 0.902
18205 measured reflections
2794 independent reflections
2369 reflections with *I* > 2σ(*I*)
*R*_int_ = 0.090

#### Refinement

*R*[*F* ^2^ > 2σ(*F* ^2^)] = 0.047
*wR*(*F* ^2^) = 0.108
*S* = 1.10
2794 reflections
208 parameters
H-atom parameters constrained
Δρ_max_ = 0.54 e Å^−3^
Δρ_min_ = −0.53 e Å^−3^

### 

Data collection: *CrystalClear* (Rigaku/MSC, 2005 ▶); cell refinement: *CrystalClear*; data reduction: *CrystalClear*; program(s) used to solve structure: *SHELXS97* (Sheldrick, 2008 ▶); program(s) used to refine structure: *SHELXL97* (Sheldrick, 2008 ▶); molecular graphics: *SHELXTL* (Sheldrick, 2008 ▶); software used to prepare material for publication: *SHELXTL*.

## Supplementary Material

Crystal structure: contains datablocks I, global. DOI: 10.1107/S1600536808042815/rn2052sup1.cif

Structure factors: contains datablocks I. DOI: 10.1107/S1600536808042815/rn2052Isup2.hkl

Additional supplementary materials:  crystallographic information; 3D view; checkCIF report

## Figures and Tables

**Fig. 1.**
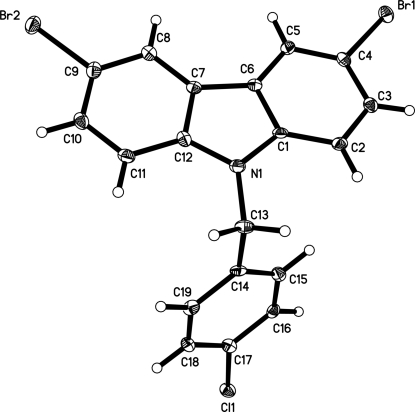
A perspective view of (I) with displacement ellipsoids drawn at the 30% probability level.

**Fig. 2.**
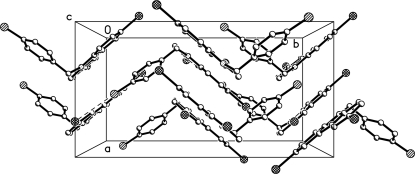
Crystal packing of (I), viewed down the a axis with H atoms omitted for clarity.

## supplementary crystallographic information

### Comment

Carbazole derivatives substituted by *N*-alkylation possess valuable pharmaceutical properties (Buu-Hoï & Royer, 1950; Harfenist & Joyner, 1983; Caulfield *et al.*, 2002; Harper *et al.*, 2002). In this paper, we report the structure of 3,6-dibromo-9-(4-chlorobenzyl)-9*H*-carbazole (I), which was synthesized by *N*-alkylation of 1-chloro-4-(chloromethyl)benzene with 3,6-dibromo-9*H*-carbazole. The carbazole ring system is essentially planar with mean deviations of 0.0277Å. The carbazole plane is inclined to the benzene ring planes at dihedral angle of 74.6 (3)°. The C—Br distances fall in the range 1.883 (4) to 1.905 (5) Å, consistent with the literature (Allen *et al.*, 1987).

### Experimental

The title compound was prepared according to the procedure of Duan *et al.* (2005*a*,*b*) from 3,6-dibromo-carbazole (Smith *et al.* 1992) and 1-chloro-4-(chloromethyl)benzene. Compound (I) (40 mg) was dissolved in mixture of chloroform (10 ml) and ethanol (5 ml) and the solution was kept at room temperature for 18 d. Natural evaporation of the solution gave colourless crystals suitable for X-Ray analysis. (m.p. 451–452 K).

### Refinement

All H atoms were included in the riding model approximation with C—H distances = 0.93 (aromatic) and 0.97 (methylene) Å, and with *U*_iso_(H) = 1.2x*U*_eq_(C).

### Crystal data

**Table:**

C_19_H_12_Br_2_ClN	*D*_x_ = 1.887 Mg m^−^^3^
*M**_r_* = 449.57	Melting point = 451–452 K
Orthorhombic, *P**b**c**a*	Mo *K*α radiation, λ = 0.71073 Å
Hall symbol: -P 2ac 2ab	Cell parameters from 5270 reflections
*a* = 8.7673 (18) Å	θ = 2.4–27.9°
*b* = 16.732 (3) Å	µ = 5.29 mm^−^^1^
*c* = 21.574 (4) Å	*T* = 113 K
*V* = 3164.8 (10) Å^3^	Prism, colorless
*Z* = 8	0.06 × 0.02 × 0.02 mm
*F*(000) = 1760	

### Data collection

**Table:**

Rigaku Saturn diffractometer	2794 independent reflections
Radiation source: rotating anode	2369 reflections with *I* > 2σ(*I*)
confocal	*R*_int_ = 0.090
ω scans	θ_max_ = 25.0°, θ_min_ = 1.9°
Absorption correction: multi-scan (*CrystalClear*; Rigaku/MSC, 2005)	*h* = −10→10
*T*_min_ = 0.742, *T*_max_ = 0.902	*k* = −19→13
18205 measured reflections	*l* = −25→25

### Refinement

**Table:**

Refinement on *F*^2^	Primary atom site location: structure-invariant direct methods
Least-squares matrix: full	Secondary atom site location: difference Fourier map
*R*[*F*^2^ > 2σ(*F*^2^)] = 0.047	Hydrogen site location: inferred from neighbouring sites
*wR*(*F*^2^) = 0.108	H-atom parameters constrained
*S* = 1.10	*w* = 1/[σ^2^(*F*_o_^2^) + (0.0488*P*)^2^ + 2.2242*P*] where *P* = (*F*_o_^2^ + 2*F*_c_^2^)/3
2794 reflections	(Δ/σ)_max_ = 0.001
208 parameters	Δρ_max_ = 0.54 e Å^−^^3^
0 restraints	Δρ_min_ = −0.53 e Å^−^^3^

### Special details

**Table:**

Geometry. All e.s.d.'s (except the e.s.d. in the dihedral angle between two l.s. planes) are estimated using the full covariance matrix. The cell e.s.d.'s are taken into account individually in the estimation of e.s.d.'s in distances, angles and torsion angles; correlations between e.s.d.'s in cell parameters are only used when they are defined by crystal symmetry. An approximate (isotropic) treatment of cell e.s.d.'s is used for estimating e.s.d.'s involving l.s. planes.
Refinement. Refinement of *F*^2^ against ALL reflections. The weighted *R*-factor *wR* and goodness of fit *S* are based on *F*^2^, conventional *R*-factors *R* are based on *F*, with *F* set to zero for negative *F*^2^. The threshold expression of *F*^2^ > σ(*F*^2^) is used only for calculating *R*-factors(gt) *etc*. and is not relevant to the choice of reflections for refinement. *R*-factors based on *F*^2^ are statistically about twice as large as those based on *F*, and *R*- factors based on ALL data will be even larger.

### Fractional atomic coordinates and isotropic or equivalent isotropic displacement parameters (Å^2^)

**Table:**

	*x*	*y*	*z*	*U*_iso_*/*U*_eq_	
Br1	0.22114 (6)	0.09041 (3)	0.56121 (2)	0.02566 (18)	
Br2	0.65417 (6)	−0.19402 (3)	0.84401 (2)	0.02964 (19)	
Cl1	0.54034 (14)	0.40983 (8)	0.94634 (5)	0.0248 (3)	
N1	0.2015 (4)	0.0764 (3)	0.83555 (17)	0.0200 (9)	
C1	0.1896 (5)	0.0868 (3)	0.7726 (2)	0.0181 (10)	
C2	0.1017 (5)	0.1422 (3)	0.7406 (2)	0.0205 (11)	
H2	0.0388	0.1783	0.7611	0.025*	
C3	0.1123 (5)	0.1410 (3)	0.6772 (2)	0.0208 (11)	
H3	0.0562	0.1771	0.6536	0.025*	
C4	0.2067 (5)	0.0860 (3)	0.6482 (2)	0.0193 (11)	
C5	0.2911 (5)	0.0299 (3)	0.6790 (2)	0.0193 (11)	
H5	0.3511	−0.0071	0.6580	0.023*	
C6	0.2827 (5)	0.0308 (3)	0.7433 (2)	0.0163 (10)	
C7	0.3559 (5)	−0.0160 (3)	0.7902 (2)	0.0170 (10)	
C8	0.4580 (5)	−0.0795 (3)	0.7888 (2)	0.0198 (11)	
H8	0.4890	−0.1020	0.7515	0.024*	
C9	0.5117 (6)	−0.1080 (3)	0.8443 (2)	0.0236 (12)	
C10	0.4625 (5)	−0.0769 (3)	0.9006 (2)	0.0240 (12)	
H10	0.4999	−0.0977	0.9375	0.029*	
C11	0.3590 (5)	−0.0155 (3)	0.9018 (2)	0.0224 (11)	
H11	0.3259	0.0052	0.9395	0.027*	
C12	0.3041 (5)	0.0154 (3)	0.8466 (2)	0.0182 (11)	
C13	0.1391 (6)	0.1292 (3)	0.8828 (2)	0.0230 (12)	
H13A	0.1194	0.0985	0.9201	0.028*	
H13B	0.0425	0.1505	0.8683	0.028*	
C14	0.2447 (5)	0.1977 (3)	0.8982 (2)	0.0190 (11)	
C15	0.2722 (5)	0.2589 (3)	0.8562 (2)	0.0208 (11)	
H15	0.2264	0.2568	0.8174	0.025*	
C16	0.3654 (5)	0.3226 (3)	0.8703 (2)	0.0198 (11)	
H16	0.3825	0.3628	0.8414	0.024*	
C17	0.4333 (5)	0.3259 (3)	0.9281 (2)	0.0220 (11)	
C18	0.4101 (6)	0.2650 (3)	0.9700 (2)	0.0237 (11)	
H18	0.4579	0.2666	1.0084	0.028*	
C19	0.3168 (5)	0.2020 (3)	0.9553 (2)	0.0214 (11)	
H19	0.3018	0.1614	0.9841	0.026*	

### Atomic displacement parameters (Å^2^)

**Table:**

	*U*^11^	*U*^22^	*U*^33^	*U*^12^	*U*^13^	*U*^23^
Br1	0.0279 (3)	0.0322 (4)	0.0169 (3)	−0.0001 (2)	0.00008 (19)	0.0030 (2)
Br2	0.0277 (3)	0.0238 (4)	0.0375 (3)	0.0038 (2)	−0.0060 (2)	0.0027 (2)
Cl1	0.0296 (7)	0.0222 (8)	0.0225 (6)	−0.0055 (5)	−0.0011 (5)	−0.0009 (5)
N1	0.025 (2)	0.018 (3)	0.0176 (19)	−0.0014 (18)	0.0005 (16)	−0.0021 (18)
C1	0.023 (2)	0.013 (3)	0.018 (2)	−0.003 (2)	−0.0011 (19)	−0.005 (2)
C2	0.019 (2)	0.016 (3)	0.027 (3)	−0.003 (2)	−0.003 (2)	−0.005 (2)
C3	0.021 (3)	0.020 (3)	0.021 (2)	−0.004 (2)	−0.004 (2)	0.001 (2)
C4	0.017 (2)	0.023 (3)	0.017 (2)	−0.004 (2)	−0.0016 (19)	0.000 (2)
C5	0.021 (2)	0.016 (3)	0.021 (2)	−0.001 (2)	0.0007 (19)	0.000 (2)
C6	0.016 (2)	0.016 (3)	0.017 (2)	−0.003 (2)	0.0019 (18)	−0.002 (2)
C7	0.017 (2)	0.015 (3)	0.019 (2)	−0.006 (2)	0.0002 (18)	−0.001 (2)
C8	0.018 (2)	0.022 (3)	0.020 (2)	−0.003 (2)	0.0014 (19)	0.001 (2)
C9	0.021 (3)	0.020 (3)	0.030 (3)	−0.003 (2)	−0.004 (2)	0.004 (2)
C10	0.027 (3)	0.023 (3)	0.022 (2)	−0.007 (2)	−0.006 (2)	0.004 (2)
C11	0.028 (3)	0.021 (3)	0.019 (2)	−0.005 (2)	−0.001 (2)	−0.003 (2)
C12	0.017 (2)	0.018 (3)	0.019 (2)	−0.008 (2)	−0.0026 (18)	0.003 (2)
C13	0.025 (3)	0.026 (3)	0.018 (2)	0.000 (2)	0.008 (2)	−0.007 (2)
C14	0.019 (2)	0.020 (3)	0.018 (2)	0.001 (2)	0.0044 (19)	−0.008 (2)
C15	0.017 (2)	0.028 (3)	0.017 (2)	0.003 (2)	0.0022 (19)	−0.002 (2)
C16	0.019 (2)	0.020 (3)	0.020 (2)	0.006 (2)	0.0042 (19)	0.002 (2)
C17	0.023 (3)	0.024 (3)	0.020 (2)	−0.001 (2)	0.000 (2)	−0.001 (2)
C18	0.032 (3)	0.024 (3)	0.015 (2)	0.003 (2)	−0.002 (2)	0.001 (2)
C19	0.027 (3)	0.016 (3)	0.021 (2)	−0.002 (2)	0.008 (2)	0.004 (2)

### Geometric parameters (Å, °)

**Table:**

Br1—C4	1.883 (4)	C8—H8	0.9300
Br2—C9	1.905 (5)	C9—C10	1.390 (7)
Cl1—C17	1.734 (5)	C10—C11	1.370 (7)
N1—C1	1.373 (6)	C10—H10	0.9300
N1—C12	1.382 (6)	C11—C12	1.384 (6)
N1—C13	1.455 (6)	C11—H11	0.9300
C1—C2	1.390 (7)	C13—C14	1.510 (7)
C1—C6	1.393 (7)	C13—H13A	0.9700
C2—C3	1.370 (6)	C13—H13B	0.9700
C2—H2	0.9300	C14—C19	1.386 (7)
C3—C4	1.387 (7)	C14—C15	1.389 (7)
C3—H3	0.9300	C15—C16	1.377 (7)
C4—C5	1.368 (7)	C15—H15	0.9300
C5—C6	1.389 (6)	C16—C17	1.383 (6)
C5—H5	0.9300	C16—H16	0.9300
C6—C7	1.431 (7)	C17—C18	1.376 (7)
C7—C8	1.390 (7)	C18—C19	1.372 (7)
C7—C12	1.402 (6)	C18—H18	0.9300
C8—C9	1.371 (6)	C19—H19	0.9300
			
C1—N1—C12	108.3 (4)	C9—C10—H10	119.9
C1—N1—C13	126.0 (4)	C10—C11—C12	119.6 (5)
C12—N1—C13	124.9 (4)	C10—C11—H11	120.2
N1—C1—C2	128.2 (4)	C12—C11—H11	120.2
N1—C1—C6	108.6 (4)	N1—C12—C11	130.7 (4)
C2—C1—C6	123.2 (4)	N1—C12—C7	109.7 (4)
C3—C2—C1	116.7 (5)	C11—C12—C7	119.6 (5)
C3—C2—H2	121.7	N1—C13—C14	112.6 (4)
C1—C2—H2	121.7	N1—C13—H13A	109.1
C2—C3—C4	119.9 (5)	C14—C13—H13A	109.1
C2—C3—H3	120.0	N1—C13—H13B	109.1
C4—C3—H3	120.0	C14—C13—H13B	109.1
C5—C4—C3	124.1 (4)	H13A—C13—H13B	107.8
C5—C4—Br1	118.3 (4)	C19—C14—C15	117.5 (5)
C3—C4—Br1	117.6 (4)	C19—C14—C13	121.0 (5)
C4—C5—C6	116.6 (5)	C15—C14—C13	121.5 (4)
C4—C5—H5	121.7	C16—C15—C14	122.0 (4)
C6—C5—H5	121.7	C16—C15—H15	119.0
C5—C6—C1	119.5 (4)	C14—C15—H15	119.0
C5—C6—C7	132.5 (5)	C15—C16—C17	119.1 (5)
C1—C6—C7	108.1 (4)	C15—C16—H16	120.5
C8—C7—C12	120.9 (4)	C17—C16—H16	120.5
C8—C7—C6	133.9 (4)	C18—C17—C16	119.9 (5)
C12—C7—C6	105.3 (4)	C18—C17—Cl1	122.1 (4)
C9—C8—C7	118.0 (5)	C16—C17—Cl1	118.0 (4)
C9—C8—H8	121.0	C19—C18—C17	120.4 (5)
C7—C8—H8	121.0	C19—C18—H18	119.8
C8—C9—C10	121.7 (5)	C17—C18—H18	119.8
C8—C9—Br2	119.0 (4)	C18—C19—C14	121.2 (5)
C10—C9—Br2	119.3 (4)	C18—C19—H19	119.4
C11—C10—C9	120.2 (5)	C14—C19—H19	119.4
C11—C10—H10	119.9		
			
C12—N1—C1—C2	−178.9 (5)	Br2—C9—C10—C11	−179.4 (4)
C13—N1—C1—C2	−8.9 (8)	C9—C10—C11—C12	−0.1 (7)
C12—N1—C1—C6	1.3 (5)	C1—N1—C12—C11	177.3 (5)
C13—N1—C1—C6	171.2 (4)	C13—N1—C12—C11	7.2 (8)
N1—C1—C2—C3	178.8 (5)	C1—N1—C12—C7	−1.8 (5)
C6—C1—C2—C3	−1.3 (7)	C13—N1—C12—C7	−171.9 (4)
C1—C2—C3—C4	0.4 (7)	C10—C11—C12—N1	−179.6 (5)
C2—C3—C4—C5	1.2 (8)	C10—C11—C12—C7	−0.5 (7)
C2—C3—C4—Br1	−177.8 (4)	C8—C7—C12—N1	−178.6 (4)
C3—C4—C5—C6	−1.8 (7)	C6—C7—C12—N1	1.7 (5)
Br1—C4—C5—C6	177.2 (3)	C8—C7—C12—C11	2.1 (7)
C4—C5—C6—C1	0.8 (7)	C6—C7—C12—C11	−177.6 (4)
C4—C5—C6—C7	−178.1 (5)	C1—N1—C13—C14	−85.7 (6)
N1—C1—C6—C5	−179.4 (4)	C12—N1—C13—C14	82.7 (6)
C2—C1—C6—C5	0.7 (7)	N1—C13—C14—C19	−111.0 (5)
N1—C1—C6—C7	−0.2 (5)	N1—C13—C14—C15	69.5 (6)
C2—C1—C6—C7	179.9 (4)	C19—C14—C15—C16	−1.0 (7)
C5—C6—C7—C8	−1.5 (9)	C13—C14—C15—C16	178.4 (4)
C1—C6—C7—C8	179.5 (5)	C14—C15—C16—C17	−0.2 (7)
C5—C6—C7—C12	178.2 (5)	C15—C16—C17—C18	1.6 (7)
C1—C6—C7—C12	−0.9 (5)	C15—C16—C17—Cl1	−176.0 (4)
C12—C7—C8—C9	−2.9 (7)	C16—C17—C18—C19	−1.7 (8)
C6—C7—C8—C9	176.7 (5)	Cl1—C17—C18—C19	175.8 (4)
C7—C8—C9—C10	2.3 (7)	C17—C18—C19—C14	0.4 (8)
C7—C8—C9—Br2	−179.1 (4)	C15—C14—C19—C18	1.0 (7)
C8—C9—C10—C11	−0.8 (8)	C13—C14—C19—C18	−178.5 (5)
